# Editorial: Bridging the gap between immunology, virology, genetics, and epigenetics in bronchiolitis: The multiomics pathway to asthma development

**DOI:** 10.3389/fimmu.2023.1154121

**Published:** 2023-02-21

**Authors:** Heidi Makrinioti, Hideaki Morita, Eleni Nastouli, Tuomas Jartti

**Affiliations:** ^1^ Department of Emergency Medicine, Massachusetts General Hospital, Harvard Medical School, Boston, MA, United States; ^2^ Department of Allergy and Clinical Immunology, National Research Institute for Child Health and Development, Tokyo, Japan; ^3^ Allergy Center, National Center for Child Health and Development, Tokyo, Japan; ^4^ Department of Infection, Immunity and Inflammation, University College London (UCL) Great Ormond Street Institute of Child Health, University College London, London, United Kingdom; ^5^ Research unit for Pediatrics, Pediatric Neurology, Pediatric Surgery, Child Psychiatry, Dermatology, Clinical Genetics, Obstetrics and Gynecology, Otorhinolaryngology and Opthalmology (PEDEGO) Research Unit, Medical Research Center, University of Oulu, Oulu, Finland; ^6^ Department of Pediatrics, Oulu University Hospital, Oulu, Finland; ^7^ Department of Paediatrics, Turku University Hospital and Turku University, Turku, Finland

**Keywords:** bronchiolitis, asthma, multiomics, mechanistic, epidemiology

Severe bronchiolitis (i.e., bronchiolitis or first episode of wheeze requiring hospitalization) during infancy is a heterogeneous condition associated with an increased risk for childhood asthma development (1, 2). Bronchiolitis cohort studies have identified early-life environmental, genetic and immune risk factors for childhood asthma development by carrying out analysis at single level (e.g., associations with respiratory virus types, host immune response or the microbiome composition of the host) (3, 4). However, severe bronchiolitis pathogenesis involves interaction of factors at multiple levels (e.g., genome, epigenome, transcriptome, metabolome, microbiome). Optimistically, the increasing use of omics methodologies in observational studies allows for a more holistic approach, that can shed light on severe bronchiolitis pathophysiology by identifying distinct biological processes associated with long-term sequelae like asthma (5). In addition, mechanistic studies are required to validate and test identified pathogenetic pathways from omics studies.

To further address the issues outlined above, our Research Topic congregates evidence from observational and interventional studies exploring the severe bronchiolitis to childhood asthma causal pathway with the aim to identify severe bronchiolitis endotypes that can guide predictive (i.e., response to treatment) and prognostic (i.e., association with long-term respiratory sequelae) enrichment strategies (see **Figure 1**). The current editorial introduces our research collection by discussing the relevant studies.

**Figure 1:**
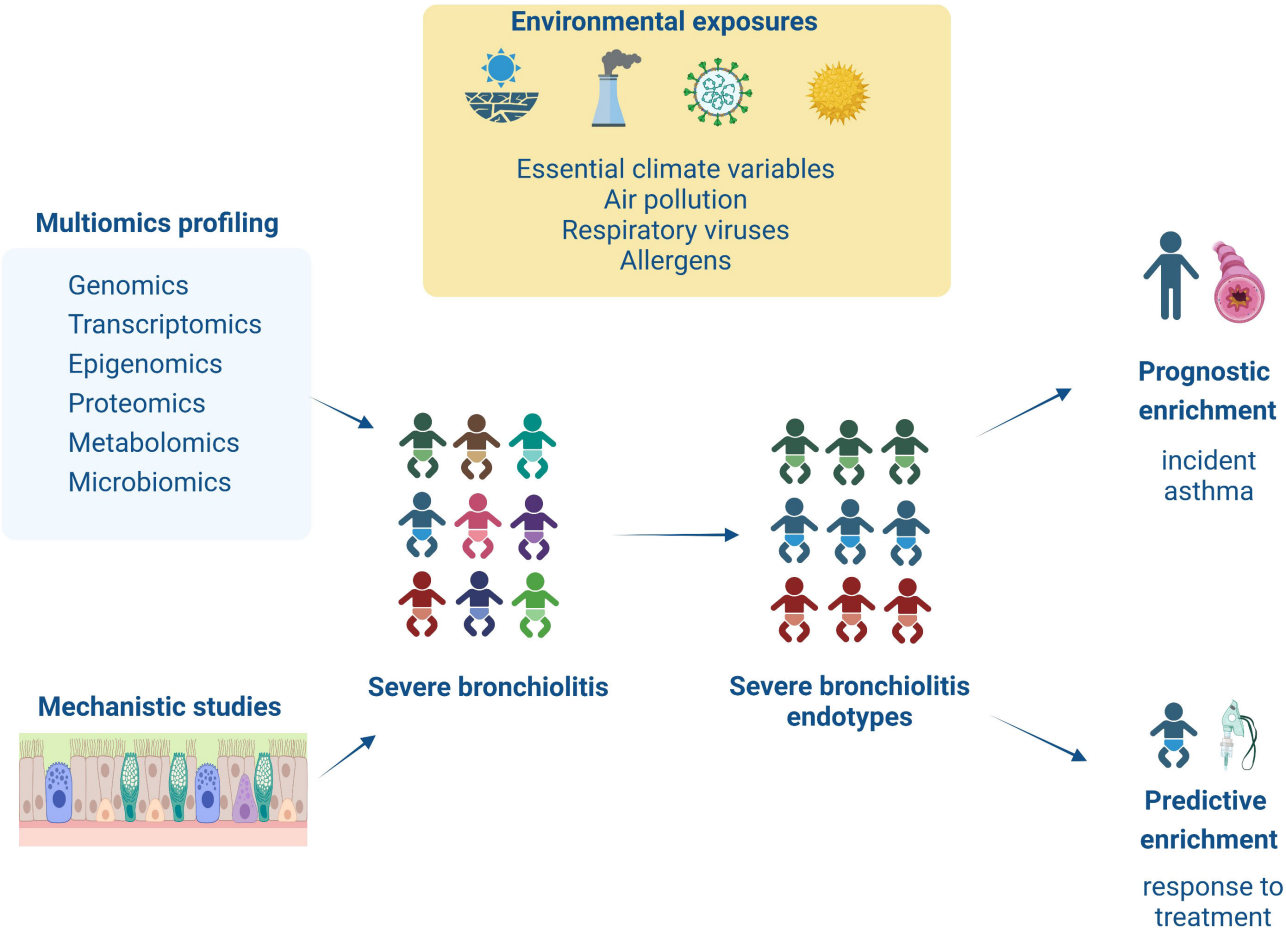
This figure summarizes the content of the articles collected on this Research Topic. Infants with severe bronchiolitis are at higher risk of having a severe and prolonged hospitalization and long-term respiratory sequelae (i.e., asthma). However, due to increased heterogeneity in clinical presentation and underlying pathology in severe bronchiolitis, we are still missing targeted treatments and sensitive biomarkers for asthma development. This Research Topic presents novel studies utilizing multiomics (i.e., genomic, transcriptomic, epigenomic, proteomic, and metabolomic) or mechanistic (i.e., *in/ex vivo*, *in vitro*) approaches to explore in-depth severe bronchiolitis pathophysiology. These findings help identify severe bronchiolitis endotypes that respond to targeted treatments (i.e., predictive enrichment) or are associated with increased risk for asthma development (i.e., prognostic enrichment).

Starting with environment-gene interactions in severe bronchiolitis, the role of epigenetic mechanisms as drivers of asthma pathogenesis has been explored (6). Epigenetic mechanisms may underpin a crosstalk between maternal and infant immune systems associated with diverse lung function trajectories (7). However, although the mediating role of epigenetic mechanisms is well understood in viral infections causing latency, further studies are needed to validate findings in viral infections causing acute illness (8). In this Research Topic, Pischedda et al. compare the methylome of children who have been hospitalized with respiratory syncytial virus (RSV)-induced bronchiolitis and are followed-up for three years. Their study assessed differentially methylated positions (DMPs) in genes in peripheral blood samples and associated with risk for recurrent wheeze development. The lead methylation position (cg24509398) identified in this study falls at the gene body of *Eyes absent protein 3* (*EYA3)*, a tyrosine phosphatase connected with pulmonary vascular remodeling, a key underlying pathogenetic mechanism in asthma. Interestingly, atopic asthma after rhinovirus-induced severe wheeze has been associated with different type of DNA methylation changes, (i.e., change in *SMAD3 gene* promoter) (9).

Keeping the above findings in mind, an *in vivo* study submitted at this research collection, focused on identifying the role of regulators *Methyl-CpG-binding domain protein 2 (MBD2)* and *Misshapen-like kinase 1 (MINK1)* in T-helper (Th)17-dominant asthma (Chen et al.). The *MBD2* and *MINK1* genes were silenced or overexpressed by small interfering RNA and plasmids and the study showed that *MBD2* and *MINK1* regulate Th17 cell differentiation and IL-17 release. It is of note that other *in vivo* models of chronic airway inflammation have showcased that Th17 responses contribute to airway remodelling too, independent of the Th2 responses (10). Through an analytical assessment of the above findings, we suggest future assessment of *EYA3* methylation patterns in Th17 cells in severe bronchiolitis as possible endotypes of early airway remodelling. The above studies identified a possible severe bronchiolitis endotype at risk of early airway remodelling. However, the question whether epigenetic changes associated with airway remodelling are triggered by specific respiratory viruses still remains unanswered.

To further approach the question around the role of respiratory viruses, two observational studies submitted at this research collection attempt to identify genetic variants and cytokine expression patterns in severe bronchiolitis and relate to subsequent asthma development (Hurme et al.; Dong et al.). The first study shows that infants with decreased expression of a thymus and activation-regulated chemokine (TARC) or increased expression of interleukin 13 (IL-13) in anti-CD3/anti-CD28 stimulated PBMC during a rhinovirus-induced acute respiratory infection are associated with relapses within a 2-month period of follow-up. The second study shows that genetic variants in the interleukin 33 gene in infants with severe bronchiolitis, regardless respiratory virus type etiology, is associated with increased risk for asthma development. Although these studies identify possible biomarkers for asthma development, we still need to understand whether pro-Th2 or Th2 mediators are more strongly associated with subsequent asthma development in comparison to respiratory virus type.

Toward this direction, a systematic review submitted at this research collection explores the potential for use of respiratory virus testing in guiding acute treatment with corticosteroids and prediction of subsequent long-term respiratory sequela (Ambrozej et al.). However, meta-analysis was not possible due to small number of studies. However, evidence points toward non-RSV viruses being associated with higher risk for asthma and increased effectiveness with corticosteroids and warrants further trials. Possibly, other than respiratory virus exposures (i.e., innate immune responses represented by macrophage differentiation or extracellular vesicles) can act as more sensitive biomarkers too. The relevant evidence is presented in the following reviews (Wang et al. and Ambrożej et al.).

In addition to respiratory virus types as possible exposures associated with increased risk for asthma development, this research collection is bringing to the fore a comprehensive review around the role of microbial dysbiosis in asthma development (Liu et al.). In response to non-protective environmental exposures (air pollutants, antibiotics, allergens), disruption of lung and gut microbiota diversity is associated with the activation of leukocytes and secretion of mediators that further promote Th2 differentiation. Therefore, as discussed at the description of this Research Topic, the interaction between exposures in severe bronchiolitis is complex, and analytical integration of data deriving from these epidemiological studies may be required.

This integrative approach is highlighted through the last study of this research collection presenting the first integration of genomic and metabolomic data in severe bronchiolitis (Ooka et al.). In this study, 749 infants with severe bronchiolitis underwent both genotyping and nasopharyngeal metabolome profiling. Through an integrated analysis, sphingomyelins, genes on chromosome 19p13 (e.g., *MUC16*), and *1,2-dioleoyl-GPG* were associated with increased risk for asthma development. Although these genes are known to be associated with asthma development, their biological functional potential is further enhanced through confirmation of metabolites expression too.

In brief, this Research Topic highlights that severe bronchiolitis is a heterogeneous condition. Different respiratory viruses, in interaction with the host genome, methylome, proteome and microbiome, can possibly drive risk for asthma development. However, the exact mechanisms are still unknown and therefore there is lack of validated predictors for severity in the acute phase as well as risk predictors for asthma. There is no doubt that, with the use of novel sampling methods and novel omics analytical approaches followed by mechanistic studies, we can advance our knowledge on bronchiolitis endotyping and identify novel therapeutic targets and biomarkers for asthma development.

## Author contributions

HMa: composed and distributed the first draft of the manuscript, designed the figure. HMo: revised the drafted manuscript and the figure. EN: revised the drafted manuscript and the figure. TJ: revised the drafted manuscript and the figure and provided detailed feedback around the included studies he co-authored. All authors contributed to the article and approved the submitted version.
